# Clinical Spectrum and Predictors of Severe Dengue Among Hospitalized Children in a Tertiary Care Hospital in Bangladesh

**DOI:** 10.7759/cureus.107326

**Published:** 2026-04-19

**Authors:** Abu Sayeed Chowdhury, Shahana Parveen, Faizul Haque Ponir, Subrata Roy, Farhana Hossain

**Affiliations:** 1 Paediatrics, Mugda Medical College and Hospital, Dhaka, BGD; 2 Paediatrics, Insaf Barakah Kidney &amp; General Hospital, Dhaka, BGD; 3 Ophthalmology, Popular Medical College, Dhaka, BGD

**Keywords:** dengue, dengue in bangladesh, pediatric dengue, predictors of severe dengue, severe dengue

## Abstract

Introduction

Dengue is a major public health concern in tropical regions, with children at increased risk of severe disease. Early identification of predictors is essential for timely intervention.

Methods

This hospital-based cross-sectional study was conducted from January to December 2024 at a tertiary care hospital in Dhaka, Bangladesh. A total of 346 children aged 0-14 years with confirmed dengue infection were included. Clinical, laboratory, radiological, and comorbidity data were analyzed. Variables associated with severe dengue were identified using the chi-square test and multivariate logistic regressio.

Results

Severe dengue was observed in 53 (15.3%) cases, with one mortality (0.3%). Common clinical features included fever in 346 (100%), vomiting in 97 (28.0%), abdominal pain in 73 (21.1%), and diarrhea in 59 (17.1%) patients. On univariate analysis, several clinical, laboratory, and radiological variables were significantly associated with severe dengue. Multivariate analysis identified elevated hematocrit (adjusted odds ratio (AOR): 9.2) as the strongest predictor, followed by pleural effusion (AOR: 7.4), ascites (AOR: 6.3), thrombocytopenia (AOR: 5.6), hypoalbuminemia (AOR: 4.8), elevated aspartate aminotransferase (AST, AOR: 4.2), and elevated alanine transaminase (ALT, AOR: 3.5).

Conclusion

Severe dengue in children is primarily associated with markers of plasma leakage and organ involvement. Early risk stratification using laboratory and radiological parameters can improve clinical outcomes.

## Introduction

Dengue is an increasingly prevalent mosquito-borne viral disease. It poses a major public health challenge in tropical and subtropical regions. Four distinct serotypes of dengue virus (DENV-1 to DENV-4) cause the disease. It is primarily transmitted by *Aedes aegypti *mosquitoes [1]. The global incidence of dengue has increased substantially over the past few decades. An estimated 50-100 million infections occur annually, placing nearly half of the world's population at risk [2].

Children are reported to have a higher risk of dengue infection and contribute substantially to the disease burden in endemic regions [3,4]. Pediatric patients more frequently require hospitalization, and severe forms, such as dengue hemorrhagic fever (DHF) and dengue shock syndrome (DSS), have been documented more commonly in this age group [3,4]. These severe manifestations are characterized by plasma leakage, bleeding, and organ dysfunction, which can lead to increased morbidity and mortality.

In Bangladesh, dengue has become an endemic disease with a rising incidence and severity. The country has experienced several major outbreaks in recent years, with the 2023 outbreak being the largest on record, reporting over 300,000 cases [5]. Ongoing surveillance data indicate a persistent and substantial disease burden.

Early recognition of severe dengue is essential. Prompt and appropriate management can lower case fatality rates to below 1% [2]. Several clinical and laboratory indicators, including gastrointestinal symptoms, hepatomegaly, thrombocytopenia, and elevated hematocrit, have been identified as predictors of severe dengue [6-8]. These predictors may differ across various populations and settings. Regional guidelines have also emphasized integrated strategies for dengue prevention, control, and clinical management in endemic settings [9].

Despite the rising burden of dengue in Bangladesh, data on the clinical features and predictors of severity among hospitalized children remain scarce. The primary objective of this study was to identify clinical, laboratory, radiological, and comorbidity-related predictors of severe dengue among hospitalized children. A secondary objective was to describe the clinical spectrum of dengue infection in this population.

## Materials and methods

Study design and setting

This hospital-based prospective cross-sectional study was conducted in the Department of Pediatrics at Mugda Medical College Hospital, Dhaka, Bangladesh, over a one-year period from January to December 2024. The primary objective of this study was to identify clinical, laboratory, radiological, and comorbidity-related predictors of severe dengue among hospitalized children, while the secondary objective was to describe the clinical spectrum of dengue infection in this population.

Study population

The study included a total of 346 children aged 0-14 years with confirmed dengue infection. In government healthcare facilities in Bangladesh, pediatric wards routinely admit patients up to 14 years of age; therefore, this age group was selected to reflect standard clinical practice.

Inclusion and exclusion criteria

The inclusion criteria are as follows: children aged 0-14 years; hospitalized patients with confirmed dengue infection (NS1 antigen and/or dengue-specific IgM antibody positive); and patients whose guardians provided informed consent.

The inclusion criteria are as follows: patients with incomplete clinical or laboratory data; patients with suspected but unconfirmed dengue infection; and patients with co-existing conditions that could confound the diagnosis of dengue severity (e.g., other acute febrile illnesses with similar clinical features).

Sample size

The sample size was estimated using the single population proportion formula:

$n = Z&sup2; &times; p &times; (1 &minus; p) / d&sup2;$

Here, Z = 1.96 is for a 95% confidence level (p = 0.15 is the expected proportion of severe dengue based on previous hospital-based pediatric studies [6,8]), and d = 0.05 (margin of error).

The calculated minimum sample size was 196. However, in this study, consecutive sampling was employed, and all eligible pediatric patients admitted with confirmed dengue during the study period were considered for inclusion.

A total of 402 children were initially admitted with dengue infection. Among them, 18 were discharged within 24 hours and were not included. Of the remaining 384 eligible patients, 28 were excluded due to a lack of parental consent. Subsequently, 10 patients were excluded due to incomplete clinical or laboratory data.

Ultimately, a total of 346 children were included in the final analysis, which exceeded the minimum required sample size.

Diagnostic criteria

Diagnosis of dengue infection was confirmed through detection of dengue NS1 antigen and/or dengue-specific IgM antibody using standard diagnostic methods.

Data collection

Data were collected using a structured form that included demographic characteristics, clinical features, comorbidities, laboratory findings, radiological parameters, and outcomes. All patients underwent detailed clinical evaluation in accordance with standard pediatric dengue assessment protocols. The variables collected were later analyzed in relation to disease severity.

Laboratory investigations

Laboratory investigations included complete blood count, hematocrit estimation, liver function tests, and serum albumin levels. Standard operational definitions were used for all clinical and laboratory variables to ensure consistency in data interpretation. Severe thrombocytopenia was defined as a platelet count <100,000/mm³ for the analysis of the association with severity. Hematocrit values were used to assess hemoconcentration, which reflects plasma leakage and disease severity. Raised hematocrit was defined as an increase of ≥20% from baseline, consistent with hemoconcentration.

Classification of disease severity

Patients were classified according to the World Health Organization (WHO) classification of dengue severity [2]. In accordance with the WHO 2009 guidelines, severe dengue was defined by the presence of severe plasma leakage (with or without shock), severe bleeding, or organ involvement. Patients who fulfilled criteria for dengue hemorrhagic fever (DHF) and dengue shock syndrome (DSS) under the earlier WHO 1997 classification were included under the category of severe dengue and analyzed together.

Statistical analysis

Initially, associations between clinical, laboratory, radiological, and comorbidity variables and disease severity were assessed using the chi-square test (univariate analysis). Variables with p-values <0.05 were subsequently included in a multivariate logistic regression model to identify independent predictors of severe dengue. Adjusted odds ratios (AOR) with 95% confidence intervals (CI) were calculated. A p-value of <0.05 was considered statistically significant. All statistical analyses were performed using Statistical Product and Service Solutions (SPSS; IBM SPSS Statistics for Windows, Armonk, NY).

Ethical considerations

This study adhered to the STrengthening the Reporting of OBservational studies in Epidemiology (STROBE) guidelines for observational studies. Ethical approval was obtained from the Institutional Review Committee of Mugda Medical College Hospital (Memo no - MuMCH-Ethi/2023/121-5). Informed consent was obtained from the guardians of all participants. Confidentiality of patient data was strictly maintained.

## Results

Study population, distribution, and outcome

A total of 346 hospitalized children aged 0-14 years were included in the study. Table 1 displays the distribution of age and sex based on disease severity. There was no statistically significant association between age group and severity (p = 0.62). A male predominance was observed (57.8%). Severe dengue was observed in 53 (15.3%) cases, while 293 (84.7%) had non-severe dengue (Table 2). One death was recorded (0.3%), while 345 (99.7%) patients recovered (Table 3).

**Table 1:** Distribution of study participants according to age group and sex in relation to dengue severity The chi-square test was used to assess the association between categorical variables and dengue severity. A p-value of <0.05 was considered statistically significant.

Variable	Severe (n=53)	Non-severe (n=293)	Total (n=346)	χ² value	p-value
Age Group				1.78	0.62
<1 year	2 (3.8%)	18 (6.1%)	20 (5.8%)		
1-5 years	15 (28.3%)	95 (32.4%)	110 (31.8%)		
6-10 years	25 (47.2%)	125 (42.7%)	150 (43.4%)		
11-14 years	11 (20.8%)	55 (18.8%)	66 (19.0%)		
Sex				0.49	0.48
Male	32 (60.4%)	168 (57.3%)	200 (57.8%)		
Female	21 (39.6%)	125 (42.7%)	146 (42.2%)		

**Table 2:** Distribution of patients according to severity of dengue DHF: dengue hemorrhagic fever; DSS: dengue shock syndrome

Severity of dengue	Number of patients (n)	Percentage (%)
Non-severe dengue	293	84.7%
Severe dengue (DHF/DSS)	53	15.3%
Total	346	100%

**Table 3:** Clinical outcomes of hospitalized children with dengue infection

Outcome	Number of patients (n)	Percentage (%)
Recovered	345	99.7%
Death	1	0.3%
Total	346	100%

Clinical features

Fever was observed in all patients (100%). The most common associated symptoms were vomiting (28%), abdominal pain (21%), and diarrhea (17%). Hepatomegaly was observed in 15% of cases. Other clinical features included bleeding manifestations (12%), body ache (11%), headache (9%), positive tourniquet test (8%), retro-orbital pain (7%), rash (7%), and seizures (0.5%) (Table 4).

**Table 4:** Frequency of clinical features among hospitalized children with dengue infection (n = 346)

Clinical features	Number of patients (n)	Percentage (%)
Fever	346	100%
Vomiting	97	28.0%
Abdominal pain	73	21.1%
Diarrhea	59	17.1%
Hepatomegaly	52	15.0%
Bleeding	42	12.1%
Body ache	38	11.0%
Headache	31	9.0%
Positive tourniquet test	28	8.1%
Rash	24	6.9%
Retro-orbital pain	24	6.9%
Seizure	2	0.6%

Comorbidities

A total of 62 (17.9%) patients had associated comorbid conditions. Among these, obesity, nephrotic syndrome, tuberculosis, leukemia, and congenital heart disease were significantly associated with severe dengue (p < 0.05). In contrast, febrile convulsion, epilepsy, autism spectrum disorder, and bronchial asthma did not show a statistically significant association (Table 5).

**Table 5:** Association between clinical and laboratory variables and severe dengue (n = 346) The chi-square test was used for variables with adequate expected cell counts, while Fisher's exact test was applied for variables with small expected frequencies. A p-value of <0.05 was considered statistically significant. For Fisher's exact test, only p-values are reported.

Variable	Severe (n=53) n (%)	Non-severe (n=293) n (%)	Test used	Test statistic	p-value
Vomiting	13 (24.5%)	35 (11.9%)	Chi-square	χ² = 4.82	0.02
Abdominal pain	11 (20.8%)	26 (8.9%)	Chi-square	χ² = 5.20	0.02
Diarrhea	11 (20.8%)	23 (7.8%)	Chi-square	χ² = 6.25	0.02
Hepatomegaly	8 (15.1%)	18 (6.1%)	Chi-square	χ² = 4.65	0.03
Minor bleeding	7 (13.2%)	15 (5.1%)	Chi-square	χ² = 3.10	0.08
Moderate-severe bleeding	4 (7.5%)	3 (1.0%)	Fisher's exact	-	<0.01
Platelet <100,000/mm³	37 (69.8%)	59 (20.1%)	Chi-square	χ² = 48.5	<0.001
Raised hematocrit	48 (90.6%)	82 (28.0%)	Chi-square	χ² = 65.2	<0.001
Pleural effusion	24 (45.3%)	6 (2.0%)	Chi-square	χ² = 92.3	<0.001
Ascites	21 (39.6%)	9 (3.1%)	Chi-square	χ² = 78.5	<0.001
ALT >1000 U/L	24 (45.3%)	35 (11.9%)	Chi-square	χ² = 22.4	<0.001
AST >1000 U/L	32 (60.4%)	53 (18.1%)	Chi-square	χ² = 33.8	<0.001
Serum albumin <3.5 g/dL	34 (64.2%)	59 (20.1%)	Chi-square	χ² = 35.7	<0.001
Age <1 year	5 (9.4%)	18 (6.1%)	Fisher’s exact	-	0.25

Univariate analysis

Table 6 displays the association between clinical features, laboratory parameters, and radiological findings with severe dengue. Among clinical variables, vomiting, abdominal pain, diarrhea, and hepatomegaly were significantly associated with severe dengue (p < 0.05). Moderate-to-severe bleeding was significantly associated with disease severity, whereas minor bleeding did not show a significant association. Laboratory parameters, including thrombocytopenia (platelet count: <100,000/mm³), raised hematocrit, elevated liver enzymes (aspartate aminotransferase (AST) and alanine transaminase (ALT): >1000 U/L), and hypoalbuminemia, were strongly associated with severe dengue (p < 0.001). Radiological evidence of plasma leakage, including pleural effusion and ascites, also demonstrated a strong association with severe dengue (p < 0.001). Age below one year was not significantly associated with disease severity (p = 0.25)

**Table 6:** Distribution of comorbidities and their association with dengue severity (n = 346) The chi-square test was used for variables with adequate expected cell counts, while Fisher's exact test was applied for variables with small expected frequencies. A p-value of <0.05 was considered statistically significant. For Fisher's exact test, only p-values are reported.

Comorbidity	Severe (n=53) n (%)	Non-severe (n=293) n (%)	Test used	Test statistic	p-value
Obesity	16 (30.2%)	4 (1.4%)	Chi-square	χ² = 48.6	<0.001
Febrile convulsion	3 (5.7%)	9 (3.1%)	Chi-square	χ² = 1.16	0.28
Bronchial asthma	3 (5.7%)	7 (2.4%)	Chi-square	χ² = 1.78	0.18
Nephrotic syndrome	3 (5.7%)	2 (0.7%)	Fisher’s exact	-	<0.01
Epilepsy	1 (1.9%)	2 (0.7%)	Fisher’s exact	-	0.40
Tuberculosis	2 (3.8%)	1 (0.3%)	Fisher’s exact	-	<0.01
Leukemia	1 (1.9%)	1 (0.3%)	Fisher’s exact	-	<0.01
Autism spectrum disorder	1 (1.9%)	2 (0.7%)	Fisher’s exact	-	0.40
Congenital heart disease	3 (5.7%)	1 (0.3%)	Fisher’s exact	-	<0.01

Multivariate logistic regression analysis

Multivariate logistic regression analysis was performed to identify independent predictors of severe dengue (Table 7). Raised hematocrit emerged as the strongest independent predictor (AOR: 9.2, 95% CI: 4.1-20.3, p < 0.001).

**Table 7:** Multivariate logistic regression analysis of the independent predictors of severe dengue (n = 346) A multivariate logistic regression model was constructed, including variables that were significant in univariate analysis. Adjusted odds ratios (AOR) with 95% confidence intervals (CI) were calculated after controlling for potential confounders. Variables with small sample sizes were included in the model; however, their estimates should be interpreted with caution due to limited statistical power.

Variable	Adjusted Odds Ratio (AOR)	95% Confidence Interval (CI)	p-value
Raised hematocrit	9.2	4.1-20.3	<0.001
Platelet <100,000/mm³	5.6	2.6-12.1	<0.001
Pleural effusion	7.4	3.2-16.5	<0.001
Ascites	6.3	2.8-13.9	<0.001
AST >1000 U/L	4.2	2.0-8.7	<0.001
ALT >1000 U/L	3.5	1.7-7.2	<0.001
Serum albumin <3.5 g/dL	4.8	2.2-10.1	<0.001
Moderate-severe bleeding	2.9	1.1-7.5	0.03
Vomiting	1.8	0.9-3.3	0.06
Abdominal pain	1.6	0.8-3.1	0.08
Diarrhea	1.5	0.7-2.9	0.1
Hepatomegaly	1.4	0.7-2.8	0.12
Age <1 year	1.2	0.5-2.7	0.25
Obesity	2.7	0.9-8.1	0.07
Nephrotic syndrome	2.4	0.4-13.5	0.32
Tuberculosis	3.1	0.4-21.8	0.26
Leukemia	2.9	0.2-39.5	0.41
Congenital heart disease	3.6	0.5-25.4	0.19

Other significant independent predictors included thrombocytopenia (platelet count: <100,000/mm³) (AOR: 5.6, p < 0.001), pleural effusion (AOR: 7.4, p < 0.001), ascites (AOR: 6.3, p < 0.001), elevated liver enzymes (AST and ALT: >1000 U/L), and hypoalbuminemia (AOR: 4.8, p < 0.001). Moderate-to-severe bleeding also remained significantly associated with severe dengue (AOR: 2.9, p = 0.03).

In contrast, clinical features, such as vomiting, abdominal pain, diarrhea, and hepatomegaly, were not independently associated with severe dengue after adjustment in the regression model. Age below one year was also an insignificant predictor.

Seasonal distribution

**Figure 1:**
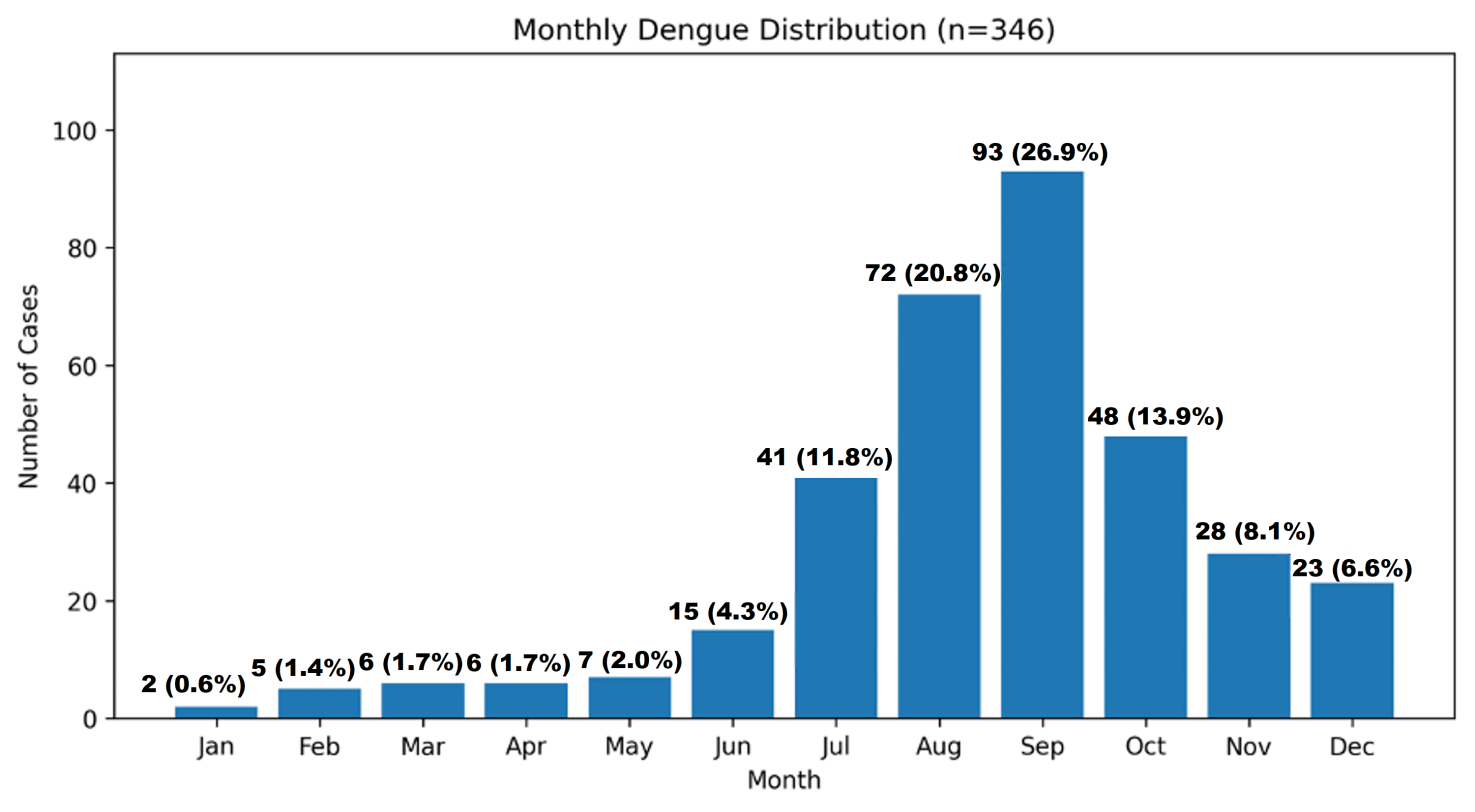
Monthly distribution of dengue cases among hospitalized children during the study period (January-December 2024)

The monthly distribution of dengue cases demonstrated a clear seasonal trend. Peak incidence was observed in September (26.9%), followed by August (20.8%) and October (13.9%). A gradual rise in cases was noted from June (4.3%) to July (11.8%). Although the majority of cases occurred during the monsoon and post-monsoon periods, dengue cases were recorded throughout the year, including winter months (Figure 1).

## Discussion

This study assessed the clinical, laboratory, radiological, and comorbidity profiles of dengue among hospitalized children and identified predictors of severe disease using both univariate and multivariate analyses. Severe dengue was observed across all age groups, with a slightly higher proportion among children aged 6-10 years; however, age was not significantly associated with disease severity. This finding aligns with previous studies reporting variable age distributions of severe dengue without consistent statistical significance [6,7]. Although infants are often considered a high-risk group due to immunological vulnerability, age under one year was not significantly linked to severity in this study, consistent with the findings from some regional studies [7,8].

A male predominance was observed in the study population, consistent with previous reports from dengue-endemic regions [3]. However, sex was not significantly associated with severity, suggesting that, while exposure patterns may differ, progression to severe disease is influenced more by biological and clinical factors than by sex alone [6,8].

A distinct seasonal pattern was observed, with the highest number of cases occurring during the post-monsoon period, especially in September. This trend aligns with previous studies showing that rainfall, temperature, and humidity significantly influence dengue transmission by affecting vector breeding and viral replication [10,11]. The occurrence of cases in winter further indicates ongoing transmission, likely driven by urbanization and Aedes mosquitoes' adaptation to indoor environments [2,12].

The proportion of severe dengue (15.3%) in this study is comparable to findings from other hospital-based pediatric studies, where severe dengue ranges from 10% to 20% [6,7]. The mortality rate was very low (0.3%), reflecting effective clinical management and early intervention, consistent with WHO reports indicating that case fatality can be reduced to below 1% with appropriate care [2].

Gastrointestinal symptoms, such as vomiting, abdominal pain, and diarrhea, were significantly associated with severe dengue in univariate analysis. Hepatomegaly was also significantly linked to disease severity. These findings align with previous studies that identified gastrointestinal involvement and hepatic dysfunction as important clinical indicators of severe disease [6,13,14]. However, in the multivariate logistic regression model, these clinical features were no longer independently significant, suggesting they may serve more as early warning signs than as definitive predictors of severity. Similar patterns have been reported in earlier research [7,14].

Bleeding manifestations showed differential associations. Moderate-to-severe bleeding was significantly associated with severe dengue, whereas minor bleeding was not. This supports previous findings that severe hemorrhagic manifestations are indicative of advanced disease and coagulopathy, while minor bleeding alone may not reliably predict severity [15].

Laboratory parameters were the most reliable indicators of severe dengue in this study. Elevated hematocrit was identified as the most significant independent predictor, indicating plasma leakage and hemoconcentration, which are central to the pathophysiology of severe dengue [2,13]. Severe thrombocytopenia, significantly increased liver enzymes, and hypoalbuminemia also demonstrated independent associations with severe dengue. These findings are consistent with previous studies identifying these parameters as predictors of severe disease [12-14].

Radiological evidence of plasma leakage, including pleural effusion and ascites, showed strong independent links with severe dengue. These findings conform to WHO classification criteria and emphasize the importance of detecting fluid buildup as a marker of disease severity [2,14]. Combining radiological findings with laboratory parameters improves the accuracy of early risk assessment.

A key strength of this study is its assessment of comorbid conditions. Tuberculosis, leukemia, nephrotic syndrome, congenital heart disease, and obesity were associated with severe dengue in univariate analysis. These results imply that pre-existing chronic illnesses and immunocompromised states may heighten the risk of severe disease. Previous research has indicated that comorbidities can affect immune response and disease progression, though data on pediatric populations remain limited [7,14,15]. Conversely, neurological and allergic conditions, such as febrile convulsions, epilepsy, autism spectrum disorder, and bronchial asthma, were not significantly linked to severity, suggesting that not all comorbidities pose the same level of risk.

The findings of this study indicate that laboratory and radiological parameters are more reliable predictors of severe dengue than clinical features alone. A combined approach that includes clinical assessment, laboratory tests, imaging findings, and evaluation of comorbidities is essential for early identification of high-risk patients and timely management. These results may help clinicians in resource-limited settings prioritize high-risk individuals. Given the observational design of this study, the findings should be interpreted as associations rather than causal relationships, and further studies are required for validation.

Limitations of the study

The single-center, cross-sectional design of this study, conducted at a tertiary care hospital, may introduce referral bias and limit the generalizability of the findings. The absence of baseline hematocrit levels required the use of population standards to define hemoconcentration, and the 2024 study period did not account for annual fluctuations in circulating dengue serotypes. Furthermore, although certain comorbidities exhibited significant associations with disease severity, the small sample sizes for conditions such as leukemia and congenital heart disease limit the statistical power in those subgroups. In addition, some comorbid conditions (e.g., obesity, nephrotic syndrome, tuberculosis, leukemia, and congenital heart disease) had small sample sizes, which may have limited the statistical power and contributed to non-significant findings in multivariate analysis. Furthermore, since this study was conducted in a single country, the results may have restricted applicability to other contexts. Furthermore, viral serotype analysis was not performed, which may influence disease severity and clinical outcomes. As this was an observational study, the findings reflect associations rather than causal relationships.

## Conclusions

Severe dengue in children remains a major clinical concern in endemic regions, such as Bangladesh. This study indicates that markers of plasma leakage and systemic involvement - particularly elevated hematocrit, thrombocytopenia, hypoalbuminemia, elevated liver enzymes, and radiological evidence of fluid accumulation - are reliable predictors of disease severity. Early detection of these indicators can enable prompt intervention and help reduce morbidity and mortality.

Incorporating these parameters into standard clinical evaluations can enhance risk stratification and optimize patient management during dengue outbreaks. These findings may also assist in developing context-specific clinical guidelines and enhancing outcomes in resource-limited settings.
